# Supplementary material for: Role of the Molecular Mass on the Elastic Properties of Hybrid Carrageenan Hydrogels
Source: Gels. 2025 Jan 20;11(1):77. doi: 10.3390/gels11010077 (PMC11765352; doi:10.3390/gels11010077)
Supplement: Supplementary file 1 [file gels-11-00077-s001.zip › gels-3407958-supplementary.pdf]

gels

Role of the Molecular Mass on the Elastic Properties of Hybrid Carrageenan Hydrogels

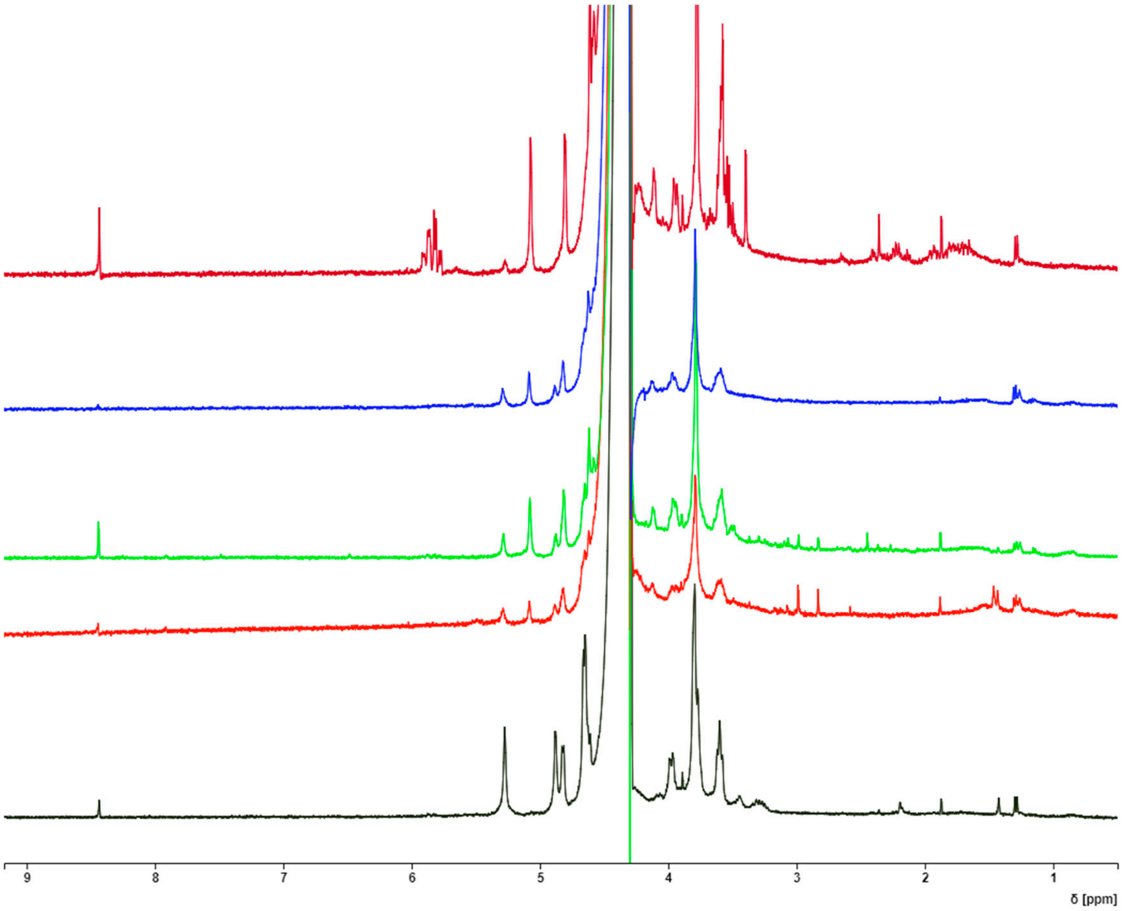

Figure S1. Proton NMR spectra of extracted and alkali-modified carrageenans. From bottom to top: *Spinosum*, *G. pistillata*, *M. stellatus*, *Gigartinale* and *Cottonii*.

 https://doi.org/10.3390/gels11010077

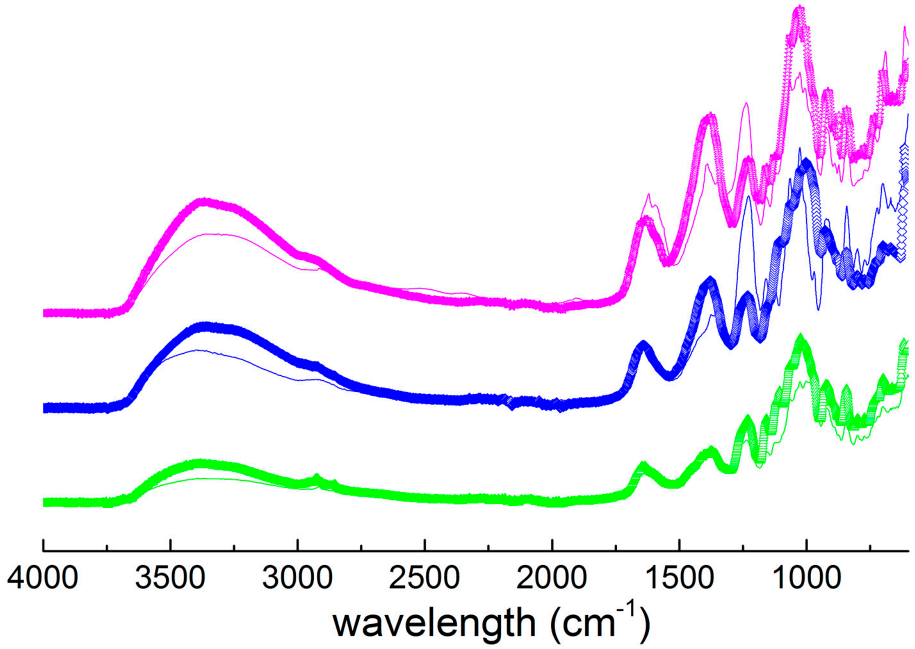

Figure S2. a. FTIR spectra of virgin (lines) and ultra-sonicated (symbols) carrageenans from (bottom to top): *M. stellatus*, Gigartinale and kappa-carrageenan.

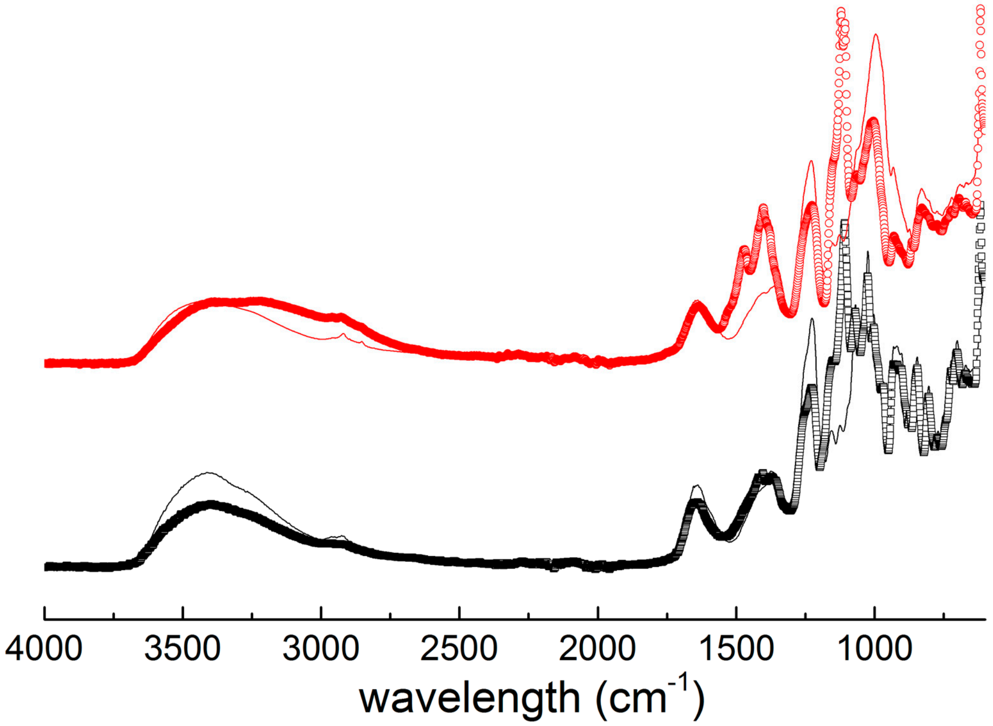

Figure S2. b. FTIR spectra of virgin (lines) and ultra-sonicated (symbols) carrageenans from (bottom to top): iota-carrageenan and *G. pistillata*. Spectra are vertically shifted to facilitate a comparison.

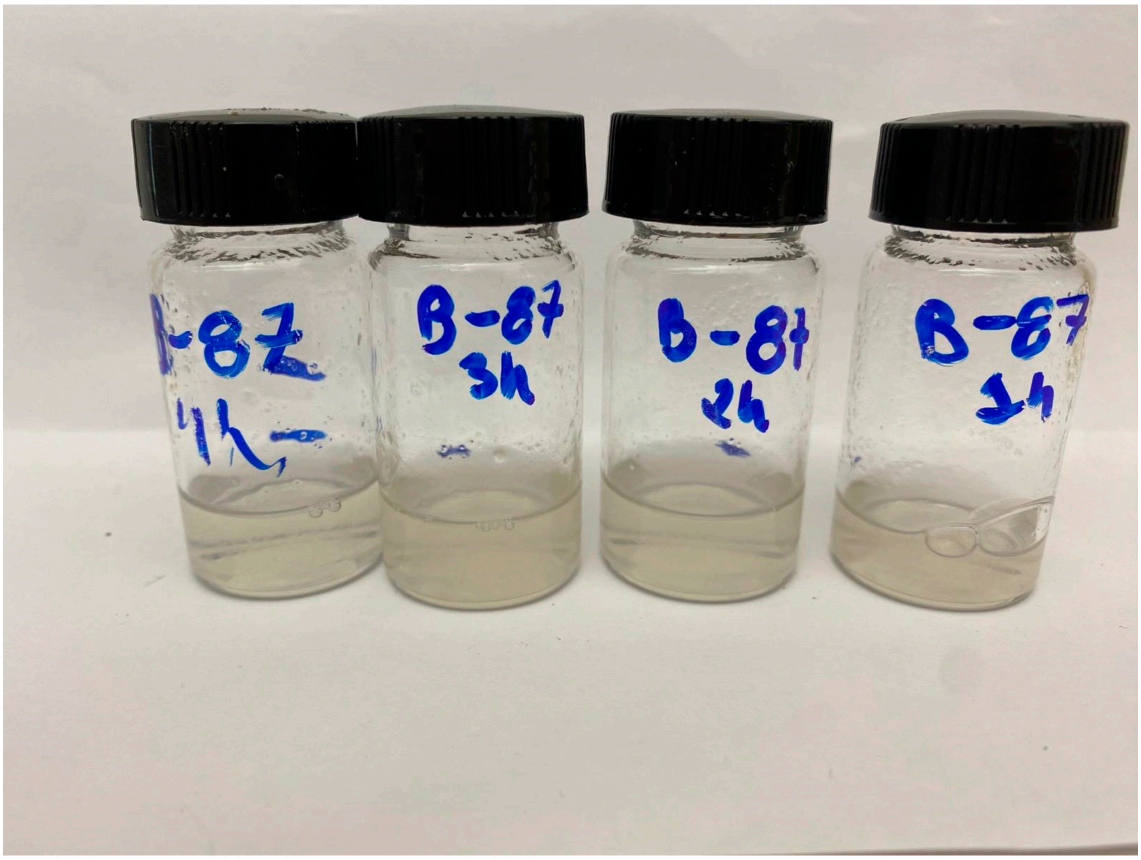

Figure S3. Iota-carrageenan aliquots sampled after different sonication times, and imaged after addition of KCl for gelling test.

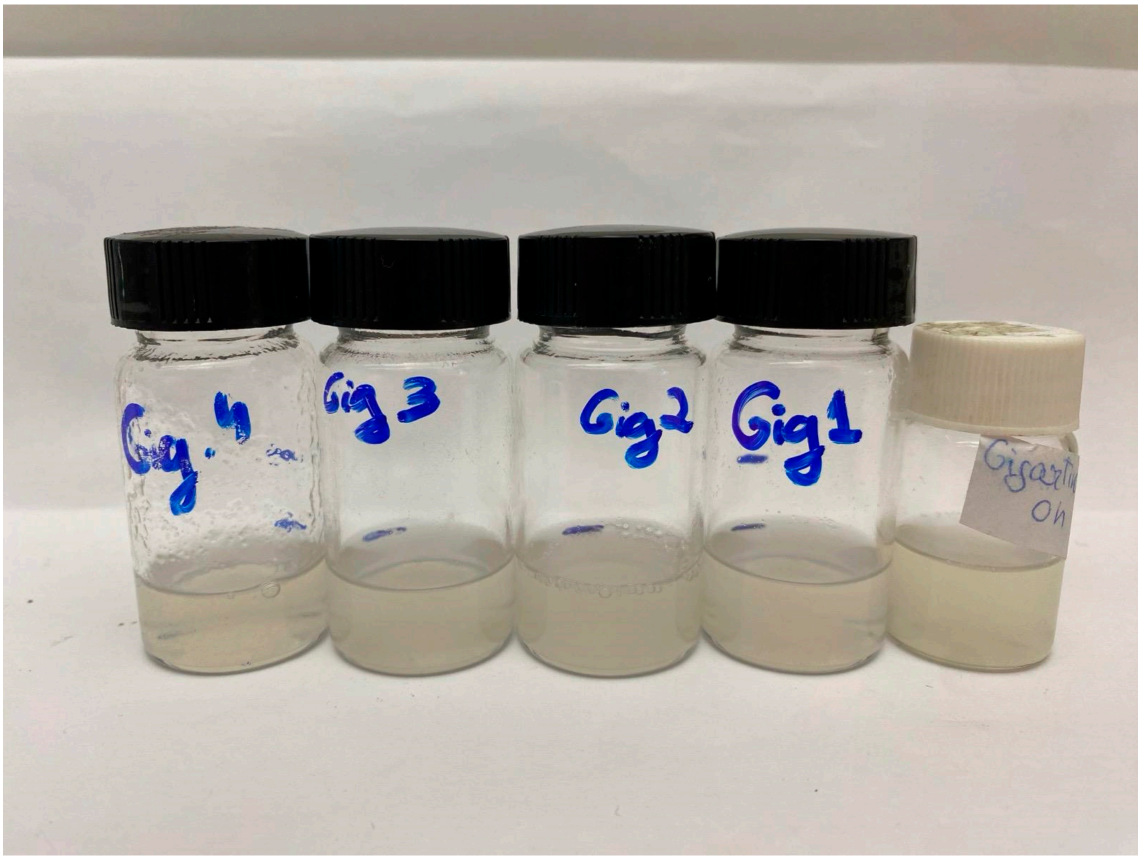

Figure S4. Carrageenan aliquots from *Gigartina pistillata* sampled after different sonication times (from right to left: 0 h, 1 h, 2 h, 3 h and 4 h), and imaged after addition of KCl for gelling test.
